# Is Neurotoxicity of Metallic Nanoparticles the Cascades of Oxidative Stress?

**DOI:** 10.1186/s11671-016-1508-4

**Published:** 2016-06-13

**Authors:** Bin Song, YanLi Zhang, Jia Liu, XiaoLi Feng, Ting Zhou, LongQuan Shao

**Affiliations:** Guizhou Provincial People’s Hospital, Guiyang, 550002 China; Nanfang Hospital, Southern Medical University, Guangzhou, 510515 China

**Keywords:** Metallic nanoparticles, Brain, Neurotoxicity, Oxidative stress, Reactive oxygen species

## Abstract

With the rapid development of nanotechnology, metallic (metal or metal oxide) nanoparticles (NPs) are widely used in many fields such as cosmetics, the food and building industries, and bio-medical instruments. Widespread applications of metallic NP-based products increase the health risk associated with human exposures. Studies revealed that the brain, a critical organ that consumes substantial amounts of oxygen, is a primary target of metallic NPs once they are absorbed into the body. Oxidative stress (OS), apoptosis, and the inflammatory response are believed to be the main mechanisms underlying the neurotoxicity of metallic NPs. Other studies have disclosed that antioxidant pretreatment or co-treatment can reverse the neurotoxicity of metallic NPs by decreasing the level of reactive oxygen species, up-regulating the activities of antioxidant enzymes, decreasing the proportion of apoptotic cells, and suppressing the inflammatory response. These findings suggest that the neurotoxicity of metallic NPs might involve a cascade of events following NP-induced OS. However, additional research is needed to determine whether NP-induced OS plays a central role in the neurotoxicity of metallic NPs, to develop a comprehensive understanding of the correlations among neurotoxic mechanisms and to improve the bio-safety of metallic NP-based products.

## Review

### Introduction

Metallic nanoparticles (NPs), with particle sizes ranging from 1 to 100 nm, possess superior physicochemical characteristics. This makes them useful in cosmetics [1], as food additives [2], in the biomedical industry [3], for environmental applications [4], and in the construction industry [5]. The widespread application of metallic NPs in many fields increases the risk human exposures. After exposure, NPs may be absorbed into the body and redistributed into secondary target organs. Numerous in vivo studies have revealed that, after animals were exposed to metallic NPs through intravenous injection [6], oral administration [7], intranasal instillation [8], and intraperitoneal injection [9], these particles can be absorbed and detected in many organs including the brain, liver, lung, spleen, and kidneys. The brain, as the most important organ, is vulnerable to the toxic effects induced by accumulated metallic NPs. Feng et al. [10] concluded that oxidative stress (OS), apoptosis, autophagy, the inflammatory response, and disturbed signaling pathways might be the main mechanisms underlying the neurotoxicity of metallic NPs. However, the interrelationships among those mechanisms remain obscure.

**Fig. 1:**
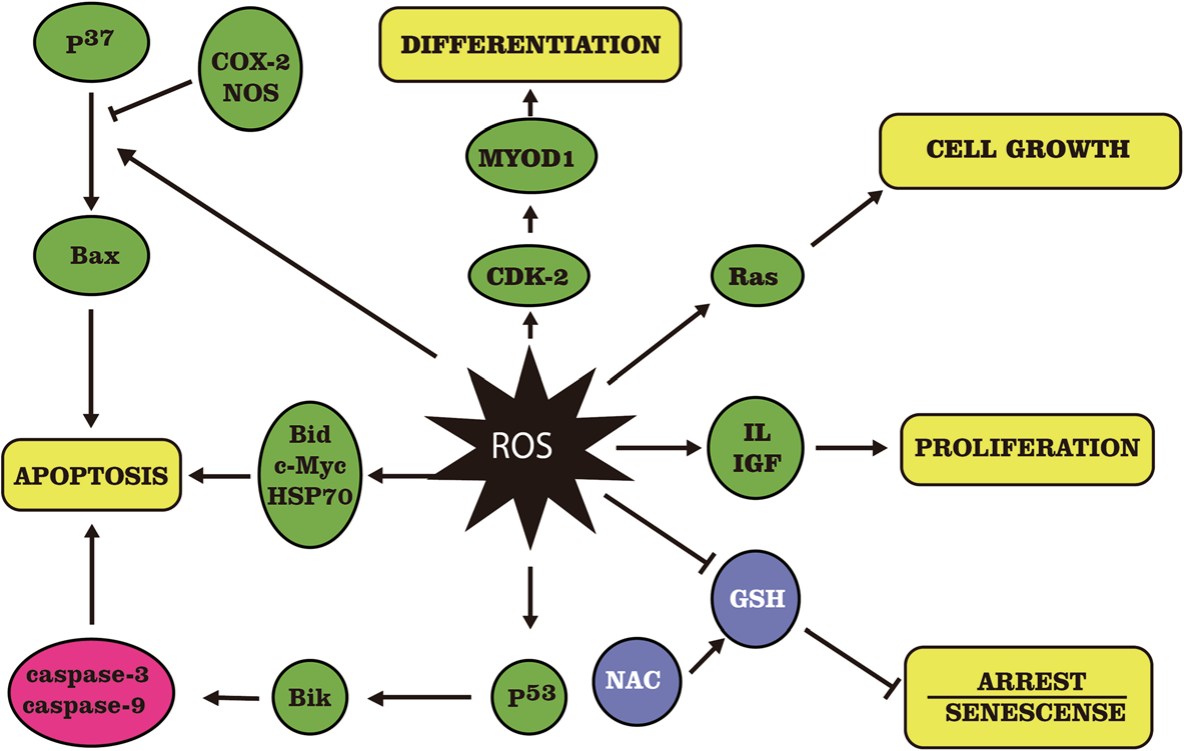
Roles of ROS in cellular responses [104]. *CDK-2* cyclin-dependent kinase 2; *COX-2* cyclooxygenase-2; *GSH* glutathione; *HSP70* 70 kDa heat shock protein; *IGF* insulin-like growth factor; *IL* interleukin; *NAC N-acety-* *l* *-cysteine*; *NF-kB* necrosis factor kappa B; *NOS* nitric oxide synthase; *ROS* reactive oxygen species

In view of the core role of OS (Fig. 1), we have summarized relevant in vivo and in vitro studies about the relationship between metallic NP-induced OS status and neurotoxicity. We conclude from available data that OS is implicated in the neurotoxicity of NPs in most situations. In addition to OS, other mechanisms are involved in the neurotoxicity of metallic NPs. Furthermore, a few rescue studies have exposed neuronal cells or animals to metallic NPs together with antioxidants. Findings from these studies show that antioxidants can reverse the neurotoxicity of metallic NPs by decreasing ROS production, up-regulating the activities of antioxidant enzymes, suppressing inflammation, and reducing the proportion of apoptotic cells. These findings suggest that the neurotoxicity of metallic NPs might involve a cascade of events following NP-induced OS. However, available data from rescue studies are insufficient to draw the definite conclusion that OS are the central mechanism of NP-induced neurotoxicity. We expect that the potential central role of OS in the neurotoxicity induced by metallic NPs might explain the complicated correlations among their neurotoxic mechanisms. However, additional rescue research is needed to determine whether OS induced by metallic NPs plays a core role in neurotoxicity.

### Applications of Metallic NPs and Their Bio-distribution in the Brain

With the rapid development of nanotechnology, metallic NPs or NP-based products, due to their outstanding physicochemical characteristics, are widely used in many fields such as cosmetics [11–13], the food industry [14–17], building materials [18, 19], biomedical applications [20–23], painting [24–26], and decontaminants [27–29]. However, widespread applications imply that humans might be unintentionally exposed to metallic NPs. After exposure, metallic NPs can be absorbed into the body and re-distributed into the main organs, possibly leading to tissue damage. Hence, metallic NP-based products become a potential threat to human health [30–32].

The brain is the most important organ, and injury to this organ is generally irreversible. Recent in vivo studies have shown that, after animals are exposed to metallic NPs such as titanium dioxide (TiO_2_), zinc oxide (ZnO), iron oxide, silica dioxide (SiO_2_), silver (Ag), or gold, these particles can enter into the body and be translocated into the brain. The limited excretion rate out of the brain leads to a gradual accumulation of metallic NPs in this organ. This, in turn, could damage neuronal cells and impair brain function, leading to permanent brain injury.

After exposure via various routes of administration, metallic NPs are translocated into the rat/mouse brain. Wu et al. [33] demonstrated that, when hairless mice were treated with TiO_2_ NPs through dermal exposure for 60 days, the Ti content in their brains was increased. Similarly, the Ti level increased in the rat brain when these animals were exposed to TiO_2_ NPs through intravenous injection [34, 35]. Female mice administered TiO_2_ NPs through intranasal instillation for 30 days exhibited an increased Ti concentration in the brain [36]. After male mice were injected intravenously with ZnO NPs, Zn ions were detected in their brain [37]. Repeated oral administration of ZnO NPs led to increased Zn ion content in the rat brain [38]. Gold NPs were detected in the brain after rats/mice were treated with gold NPs through intravenous injection [6, 39] or inhalation [40]. The Ag content in the brain increased after rats were exposed to Ag NPs through subcutaneous injection [41], intravenous injection [42, 43], oral gavage [44], other oral exposure [7, 45], or intranasal instillation [8]. The Ag level in the brain increased when mice were exposed to silver NPs through intraperitoneal injection [9], repeated oral administration [46], or intravenous injection [47]. Rabbits that were treated with Ag NPs intravenously demonstrated increased Ag content in the brain [48]. TiO_2_ and silica NPs even passed the placental barrier to accumulate in the fetal brain when pregnant mice were exposed to TiO_2_ NPs [49], which suggested a potential for neurodevelopmental toxicity.

TiO_2_ and Ag NPs are employed frequently to examine the bio-distribution of metallic NPs after systematic administration. In order to fully illustrate how metallic NPs are absorbed into the body, translocated into the brain, and excreted from the brain, more relevant research that employs different metallic NPs besides TiO_2_ and Ag is needed. In addition, the potential for neurodevelopmental toxicity of metallic NPs should be investigated.

### The Role of OS Induced by Metallic NPs in Neurotoxicity

#### Brief Description of Oxidative Stress and its Relationship with Brain Disorders

OS can be defined as disturbed redox homeostasis caused by excessive reactive oxygen species (ROS) or/and reactive nitrogen species (RNS) production, or decreased activities of antioxidant enzymes in response to harmful stimuli. Excessive ROS and RNS production can, in turn, damage DNA (determined by measuring the level of 8-hydroxy-2′-deoxyguanosine), oxidize proteins (determined by measuring the level of carbonyls), and induce lipid peroxidation, all of which can lead to tissue damage. In the process of OS, the activities of antioxidant enzymes such as superoxide dismutase (SOD), catalase (CAT), and glutathione peroxidase (GSH-Px), are inhibited in most situations. Meanwhile, non-enzymatic antioxidants such as vitamin C, vitamin E, and glutathione (GSH), are also depleted [50].

Lipids are abundant in brain tissue, and oxygen consumption in the brain accounts for nearly a quarter of the whole body’s consumption. Hence, the brain is more sensitive to hypoxic injury than other tissues and is vulnerable to oxidative damage. The pathology of neurodegenerative diseases [51–53] such as Alzheimer’s disease, Parkinson’s disease, and psychiatric disorders [54–56] (e.g., anxiety, autism, major depression) are closely related to the OS status in the brain. Meanwhile, environmental stimuli, such as air pollution, can induce oxidative damage in the brain, potentially leading to neurodegenerative diseases [57, 58].

OS is involved in heavy metal-induced neurotoxicity [59–61]. Metallic NPs, as another type of “environmental stimuli,” also affect the OS status in the brain. Recent studies have revealed that OS is implicated in the neurotoxicity of metallic NPs [62, 63]. In addition to oxidative stress, the inflammatory response, apoptosis, autophagy, and cell signaling pathways are the main mechanisms underlying the neurotoxicity of metallic NPs [10]. However, the correlations among these mechanisms are complex. It is possible that one mechanism plays a dominant role in the neurotoxicity of metallic NPs. In view of the pivotal role of OS in brain disorders, we have summarized relevant in vivo and in vitro published articles dealing with the correlations between metallic NP-induced OS and neurotoxicity.

#### In Vivo Studies About the Involvement of OS in the Neurotoxicity of Metallic NPs

TiO_2_ NPs impair mitochondrial functions and lead to OS in the rat and mouse brain [64, 65]. Although TiO_2_ NPs could not be detected in the brain zones after mice were exposed through nasal instillation, the activities of SOD, CAT, GSH-Px, and acetylcholine esterase were inhibited in the brain, probably indirectly [66]. After mice were administered TiO_2_ NPs orally, OS biomarkers showed differentiated responses. Although the activities of SOD and GSH-Px in the cortex and hippocampus were inhibited, levels of malondialdehyde (MDA; an index of lipid peroxidation) and ROS production remained unaffected [67]. Ze et al. [68] treated mice with three doses of TiO_2_ NPs nasally for 90 days and found that the levels of superoxide (O_2_^−^), H_2_O_2_, MDA, protein carbonyls, and 8-hydroxy-2′-deoxyguanosine in the mouse brain were increased in all groups compared with control animals. Furthermore, microarray analyses showed that the expression of OS-related genes in the mouse brain was also changed.

Inhalation exposure of mice to TiO_2_ NPs increased the brain levels of H_2_O_2_ and MDA [69]. After Meena et al. [70] injected rats with TiO_2_ NPs intravenously, the Ti content in the brain increased, leading to excessive ROS production and MDA, accompanied by inhibited activities of SOD and GSH-Px. In addition to OS, the proportion of apoptotic cells increased and the expression of nuclear factor-kB (NF-kB), p38, nitric oxide, interferon-γ, and tumor necrosis factor-α in the brain were elevated. Based on those findings, they concluded that TiO_2_ NP-induced OS in the rat brain might lead to inflammation and apoptosis, which contributed to the neurotoxicity of TiO_2_ NPs. Hu et al. [71] also reported that, after exposure to TiO_2_ NPs, the Ti content in the mouse brain increased, inducing ROS production and inhibiting antioxidant activities in the hippocampal areas, and increasing the proportion of apoptotic cells. They concluded that apoptosis was initiated by NP-induced OS in the brain. After rats were administered TiO_2_ and Ag NPs through a single injection, there was a change in the expression of OS-related genes in the rat brain [72]. At the same time, the antioxidant capability as well as the renin-angiotensin system in the brain was disrupted.

Ag NPs up-regulated heme oxygenase-1 (HO-1) expression at both the gene and protein levels in the hippocampus, but not in the cortex, after mice were exposed through intranasal instillation [73]. Another study using microarray analyses [74] found that, after mice were treated with Ag particles (25 nm), the expression of 18 genes in the caudate nucleus, 14 in the frontal cortex, and 29 in the hippocampal area were altered. Ag NPs (23 and 29 nm) administered by intraperitoneal injection for 7 days inhibited the activities of SOD and GSH-Px and increased MDA production in the temporal cortex of rats. In addition, the short-term memory of rats was impaired, and they performed poorly in behavioral tests [75]. These findings suggested that the expression of OS-related biomarkers in response to NPs might be regionally specific in the brain.

OS, determined by increased ROS production and decreased expression of CuZn-SOD and Mn-SOD, was induced in the mouse brain after administration of TiO_2_, ZnO, or Al_2_O_3_ NPs [76]. In another study, ZnO NPs inhibited the activities of SOD and GSH-Px and increased the MDA content in the mouse brain after intraperitoneal injection [77]. These effects appeared to contribute to impaired learning and memory ability.

Wu et al. [78] found that, after rats received SiO_2_ NPs through intranasal instillation, oxidative damage that induced an inflammatory response and disturbed neurotransmitters was observed in the striatum. They confirmed those findings in vitro by exposing rat pheochromocytoma cell line (PC12) to SiO_2_ NPs and showing that excessive ROS caused by the NPs was accompanied by increased apoptosis and a decreased number of cells in the G2/M phase of the cell cycle through the p53-mediated signaling pathway and reduced dopamine production. The same research group conducted another in vivo and in vitro study to examine the neurotoxicity of iron oxide NPs. They found that, after rats received iron oxide NPs through intranasal instillation, the expression of OS-related biomarkers in the brain showed region-specific changes. Although the GSH content in the striatum was increased, it remained unchanged in the hippocampus. H_2_O_2_ levels were elevated in the striatum and hippocampus, but SOD activity and MDA levels were unaffected in these areas. Findings obtained from PC12 cells exposed to iron oxide NPs were consistent with their previous research [79].

Parveen et al. [80] exposed rats to silica NPs through intranasal instillation and discovered that the silica content in the rat corpus striatum was elevated. This accumulation increased levels of H_2_O_2_, O_2_^−^, and protein carbonyls, inhibited activities of SOD, GSH-Px, and CAT, and decreased the GSH level in the rat corpus striatum. Meanwhile, the expression of genes and proteins related to apoptosis, such as bax, p53, bcl-2, and cytochrome c, was changed in the rat corpus striatum. Together, these findings indicated that silica NP-induced OS in the rat corpus striatum might lead to apoptosis, which contributed to the poor performance of animals in behavioral tests.

Although OS is clearly implicated in the neurotoxicity of metallic NPs, how these NPs regulate the OS status in the brain remains unclear. Ze et al. [81] reported that TiO_2_ NPs were detected in the mouse brain after intranasal instillation. This accumulation induced OS in the mouse brain that was characterized by excessive levels of H_2_O_2_, O_2_^−^, MDA, protein carbonyls, and 8-hydroxy-2′-deoxyguanosine. TiO_2_ NP-induced OS contributed to spongiocyte proliferation and hemorrhage in the mouse brain. Further experiments showed that the expression of p38, Jun *N*-terminal kinase, NF-kB, nuclear factor-2 (Nrf-2), and HO-1 was up-regulated. This suggested that oxidative impairments were probably mediated through the p38-Nrf-2 signaling pathway. Other studies revealed that OS can be mediated by Nrf-2 [82, 83]. More research is needed to investigate comprehensively how metallic NPs mediate OS in the brain.

#### In Vitro Studies About the Involvement of OS in the Neurotoxicity of Metallic NPs

Long et al. [84, 85] demonstrated that TiO_2_ NPs increased the levels of ROS, H_2_O_2_, and O_2_^−^ in BV2 cells (an immortalized mouse microglial cell line). These NPs also increased ROS production in the primary astrocytes as well as induced mitochondrial dysfunction and altered mitochondrial morphology, leading to decreased cell viability [86]. Wu et al. [87] discovered that TiO_2_ NPs reduced the viability of PC12 cells, enhanced production of ROS and MDA, decreased GSH levels, and inhibited SOD activity. They concluded that NP-induced OS reduced the mitochondrial membrane potential, induced apoptosis, and inhibited the cell cycle. Kim et al. [88] showed that OS and DNA damage were involved in the toxic effects of silica NPs on human neuronal cells (SH-SY5Y). After Yang et al. [89] exposed SK-N-SH (human neuroblastoma cell line) and neuro2a (mouse neuroblastoma cell line) cells to silica NPs, they found that ROS production was enhanced and cell viability reduced in both cell lines. In another study, silica NPs increased the production of ROS, RNS, and IL-1β in rat primary microglial cells [90]. Similarly, mesoporous silica NPs increased the production of ROS and MDA and decreased the level of GSH-Px in PC12 cells [91].

Ag NPs increased ROS production and up-regulated the expression of OS-related genes, such as those encoding HO-1 and matrix metalloproteinases-3, in PC12 cells in a size- and dose-dependent way. Apoptosis was also observed [92]. Kim et al. [93] found that, after primary cerebral cortical neurons were exposed to Ag NPs, cell viability was reduced, ROS production was elevated, and the proportion of apoptotic cells was increased. This indicated that NP-induced OS led to cell apoptosis. Copper oxide NPs reduced the viability of primary rat brain astrocytes and enhanced ROS production [94]. Xu et al. [95] reported that copper NPs reduced the viability of PC12 cells, increased ROS production, decreased SOD activity, and enhanced the proportion of apoptotic cells. Wang et al. [96] demonstrated that PC12 cells incubated with manganese, Ag, or copper NPs exhibited alterations in the expression of dopaminergic system-associated genes. At the same time, copper NPs up-regulated the expression of thioredoxin reductase 1. Down-regulated GSH-Px expression was detected in the copper and Ag NPs groups. Manganese NPs did not change the expression of thioredoxin reductase 1 or GSH-Px. These findings indicated that the expression of OS-related biomarkers was differentiated when neuronal cells were exposed to different metallic NPs.

Iron oxide NPs increased ROS production in SH-SY5Y cells. This was accompanied by impaired mitochondrial function and an increased proportion of apoptotic cells [97]. However, ROS production was not enhanced by iron oxide NPs in oligodendroglial cell lines [98]. However, it is not possible to conclude that OS was not induced because other OS-related biomarkers were not assessed.

Gold NPs induced ROS in the C17.2 neural progenitor cell line in a dose-dependent manner [99]. Although gold NPs did not induce cytotoxicity in human astrocytes, they increased ROS production, up-regulated the activity of NF-kB, and reduced micronuclei formation [100]. After Sruthi et al. [101] treated C6 cells (a rat glial cell line) with ZnO NPs for 3 and 6 h, ROS production was enhanced. However, after a 24-h exposure, the ROS levels in ZnO NP-treated cells decreased to the control group level. Additional studies examining other OS-related biomarkers, such as SOD and GSH-Px, are needed to further assess the NP-induced OS status in this system. Recently, zirconium oxide NPs were reported to reduce cell viability, enhance the production of ROS and MDA, reduce GSH levels, and induce genotoxic effects in the PC12 and N2a cell lines [102].

Huerta-Garcia et al. [103] found that TiO_2_ NPs-induced changes in ROS production and the activities of antioxidant enzymes in C6 and U373 (human glial cell) cells were not always consistent. Thus, TiO_2_ NP-induced OS is complicated and may be associated with exposure time. Future research should focus on the correlation between exposure time and metallic NP-induced OS.

**Fig. 2:**
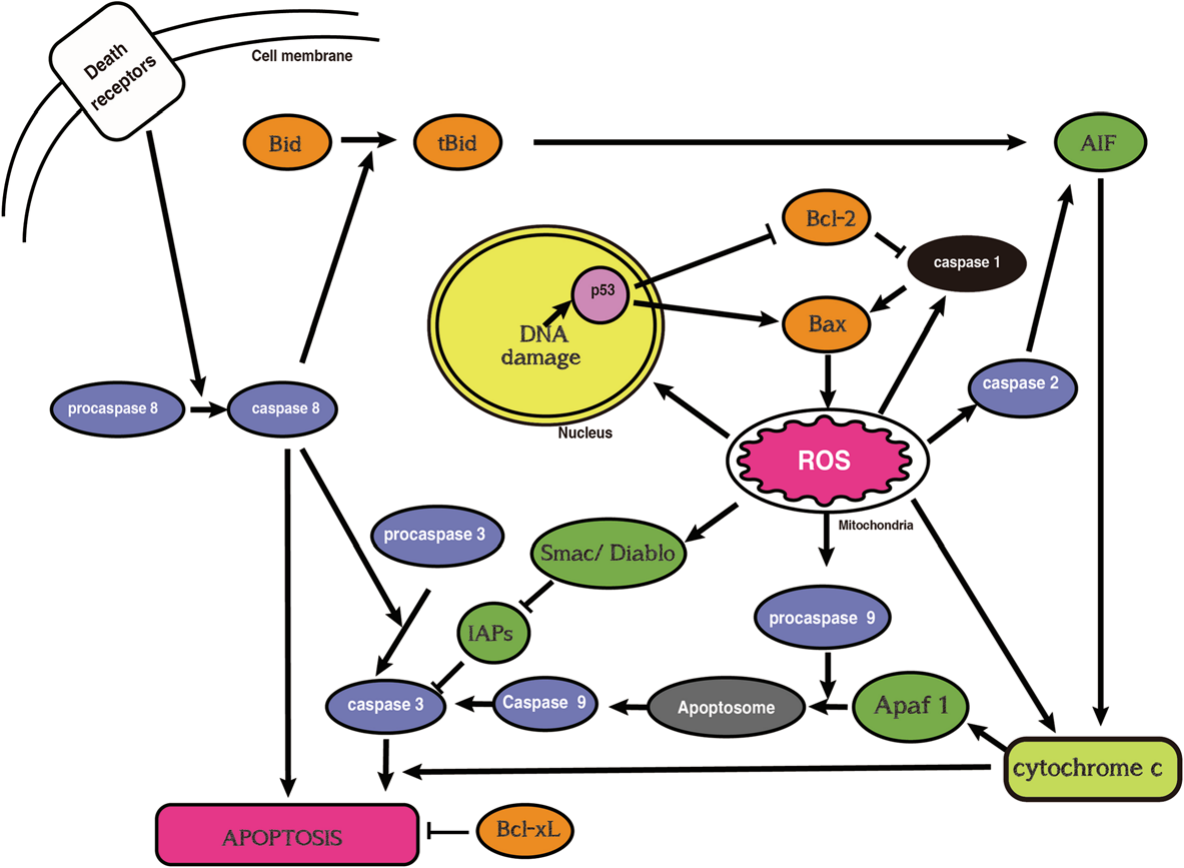
Schematic representation of apoptosis signals induced by ROS [104]. *AIF* apoptosis-inducing factor; *Apaf-1* apoptotic protease activating factor 1; *DISC* death-inducing signaling complex; *ROS* reactive oxygen species; *TRAIL* tumor necrosis factor-alpha-related apoptosis-inducing ligand

**Fig. 3:**
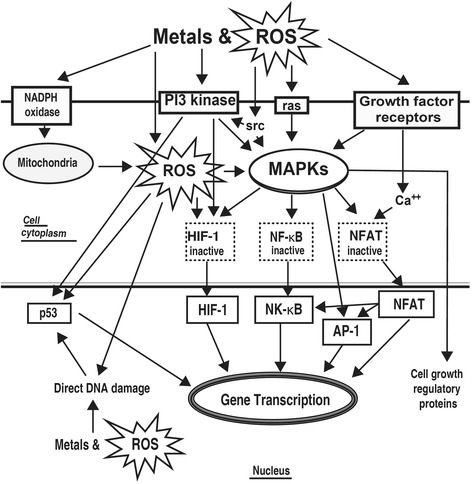
Signaling pathways activated by ROS [127]. *ROS* reactive oxygen species; *NADPH* reduced nicotine adenine dinucleotide phosphate; *MAPK* mitogen-activated protein kinase; *HIF-1* hypoxia-inducible factor 1; *NF-kB* necrosis factor kappa B; *NFAT* nuclear factor of activated T cells; *AP-1* activator protein-1

**Fig. 4:**
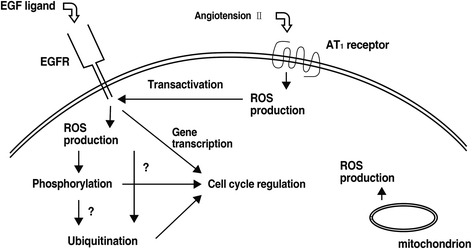
The effects of ROS on cell cycle regulation [106]. *EGFR* epidermal growth factor receptor; *EGF* epidermal growth factor; *ROS* reactive oxygen species

**Fig. 5:**
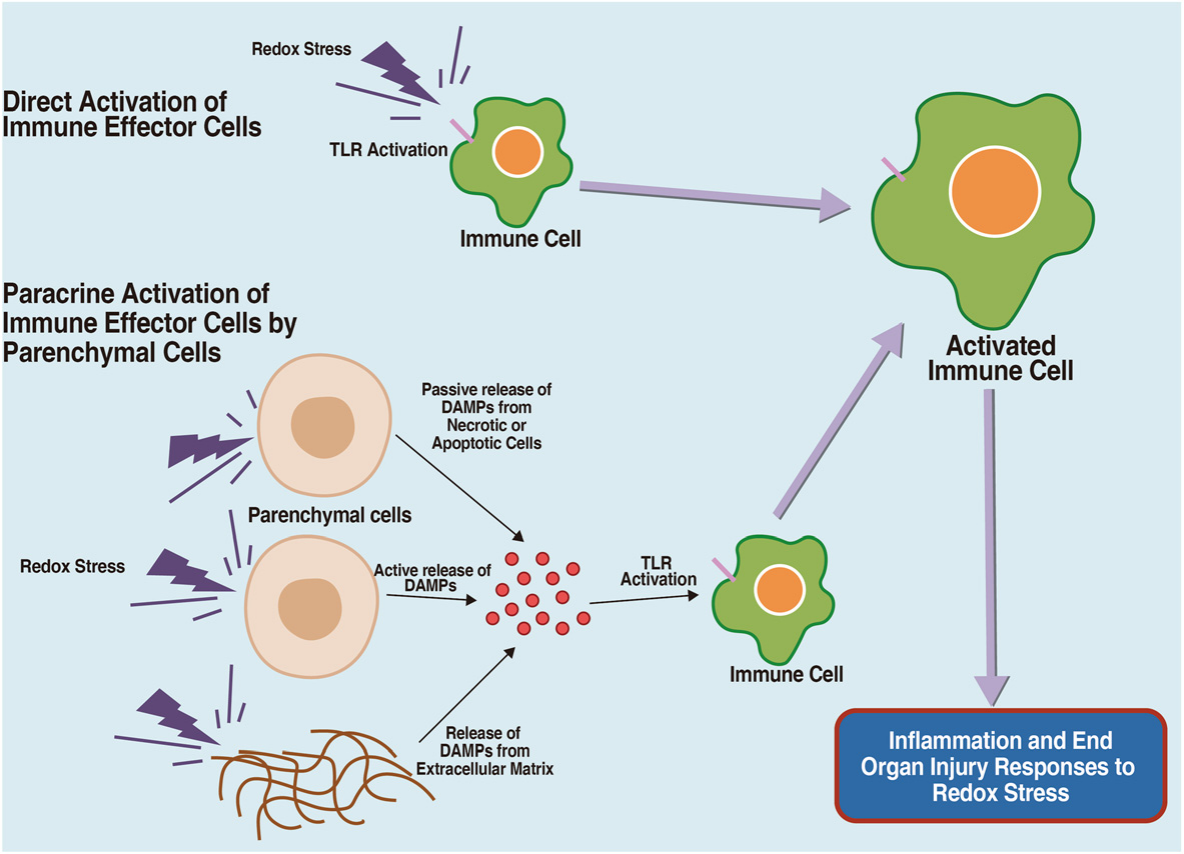
Inflammatory response mediated by OS [107]. *DAMPs* damage-associated molecular pattern molecules; *TLR* toll-like receptor

Findings from the abovementioned studies suggested that OS was involved in the neurotoxicity of NPs in most situations. Although numerous studies have shown that OS can increase apoptosis (Fig. 2) [104], activate signaling pathways [105] (Fig. 3), affect cell cycling (Fig. 4) [106], and induce inflammation (Fig. 5) [107], it is still not possible to draw the definite conclusion that the cellular responses involved in the neurotoxicity of metallic NPs are mediated by NP-induced OS.

#### Rescue Studies About the Involvement of OS in the Neurotoxicity of Metallic NPs

Rescue studies examining the role of OS in the neurotoxicity of NPs might help determine whether the neurotoxicity of metallic NPs involves a cascade of events following NP-induced OS. *N*-acetyl-l-cysteine (NAC) exhibited both antioxidant and neuroprotective capabilities and decreased the production of ROS induced by ZnO NPs in rat primary astrocytes [108–110]. At the same time, the Jun N-terminal kinase signaling pathway was suppressed, mitochondrial impairment was relieved, and the proportion of apoptotic cells was decreased in cells pretreated with NAC 6 h before NP exposure. In another study [111], NAC reversed the elevated proportion of apoptotic cells induced by ZnO NPs in U87 human glial cells. These findings suggested that NP-mediated OS activated cell signaling pathways, mitochondrial injury, and apoptosis. ZnO NPs reduced cell viability, enhanced ROS production, increased the glutathione disulfide level, inhibited GSH-Px activity, and increased apoptosis in SH-SY5Y cells. Those toxic effects were reversed by pretreating cells with NAC or esculetin [112]. Esculetin possesses antioxidant properties [113–115].

Chlorophyllin is an effective ROS scavenger. It also possesses antioxidant properties [116–118]. DNA damage, determined by the comet assay, was detected in the mouse brain after mice were exposed to TiO_2_ NPs. This damage was prevented by co-treatment with chlorophyllin [119]. This indicated that NP-induced OS can lead to DNA damage. Niska et al. [120] found that, after HT22 cells were incubated with copper NPs, cell viability was reduced, the activities of GSH-Px and SOD were inhibited, ROS production was increased, and the proportion of apoptotic cells was elevated. However, pretreating cells with crocetin (an antioxidant with neuroprotective capabilities that can counteract OS [121–123]) 1 h before NP exposure prevented those changes. Pretreating PC12 cells with *N*-(mercaptopropionyl)-glycine (another type of ROS scavenger [124]) inhibited the apoptosis induced by TiO_2_ [125] and ZnO NPs [126]. The reduced cell viability caused by TiO_2_ NPs was also ameliorated [125]. These findings suggested that NP-induced OS can lead to cell apoptosis.

#### Brief Summary

The findings described in this review support the conclusion that OS is involved in the neurotoxicity of metallic NPs. However, the NP-induced OS status was mainly assessed by measuring ROS production and the activities of antioxidant enzymes. Including measurements of RNS production and levels of non-enzymatic antioxidants would provide an improved basis for assessing the NP-induced OS status comprehensively.

Other studies reviewed here implicate apoptosis, inflammation, and cell cycle arrest in the neurotoxicity of metallic NPs. Findings from a few rescue studies suggest that pretreatment or co-treatment with antioxidants can inhibit the inflammatory response, reduce the proportion of apoptotic cells, and reverse NP-induced neurotoxicity. These findings indicate that NP-induced OS might be a central mechanism underlying the neurotoxicity of metallic NPs. However, more rescue research studies are needed to understand the core role of OS in the neurotoxicity of metallic NPs.

## Conclusions

With the widespread application of metallic NP-based products, the toxic effects induced by these particles have become a significant threat to brain health. Relevant studies have revealed that OS and other mechanisms, such as apoptosis and the inflammatory response, are involved in the neurotoxicity of metallic NPs. However, correlations among these mechanisms are unclear and do not fully support causality. In view of the purported central role of OS, a few recent rescue studies pretreating neuronal cells or co-treating animals, with antioxidants suggest that the neurotoxicity of metallic NPs might involve a cascade of events triggered by OS.

Based on the potentially pivotal role of OS in the neurotoxicity of metallic NPs, here are some suggestions for future research:

1. The bio-distribution of different metallic NPs should be investigated comprehensively
2. More rescue research is needed to ascertain the core role of OS
3. The mechanisms by which metallic NPs trigger OS and how NP-induced OS mediates other mechanisms of neurotoxicity should be studied in detail
4. The correlation among OS status, OS-related biomarkers, and biological effects induced by NPs should be explored
5. NP-induced OS status should be investigated in more detail by assessing multiple biomarkers such as the production of both ROS and RNS, activities of antioxidant enzymes, and the levels of non-enzymatic antioxidants
6. Because OS-related biomarkers are probably region-specific in the brain, it is inappropriate to measure biomarkers in whole brain tissue
7. Additional comparisons about the OS status in the brain induced by different metallic NPs are needed
8. Changing the physicochemical property of metallic NPs to inhibit NP-induced OS should be investigated as a feasible means for reducing their neurotoxicity

Overall, we expect that in-depth investigations of the central role of OS in metallic NP-induced neurotoxicity will help define how best to prevent this toxicity and can help us unravel the complicated correlations among neurotoxic mechanisms of metallic NPs.
